# Datation de la grossesse en pratique courante au Cameroun: fiabilité de la date de dernières règles

**DOI:** 10.11604/pamj.2014.17.264.3692

**Published:** 2014-04-11

**Authors:** Jean Dupont Kemfang Ngowa, Emmanuel Mando, Emilienne Guegang, Anny Ngassam, Jean Marie Kasia

**Affiliations:** 1 Service de Gynécologie/ Obstétrique, Hôpital Général de Yaoundé, BP: 5408, Yaoundé, Cameroun; 2 Departement de Gynécologie / Obstétrique, Faculté de Médecine et des Sciences Biomédicales, Université de Yaoundé I, BP: 1364, Yaoundé, Cameroun; 3 Service de Radiologie, Hôpital Général de Yaoundé, BP: 5408, Yaoundé, Cameroun

**Keywords:** Date de dernières règles, échographie de datation, datation de la grossesse, Yaoundé, last menstrual period, dating ultrasound, pregnancy dating, Yaoundé

## Abstract

**Introduction:**

La détermination précise de l’âge gestationnel (AG) est essentielle pour un suivi adapté de la grossesse. La date de dernières règles (DDR) et l’échographie de datation du premier trimestre sont les moyens habituels de datation de la grossesse. La DDR est souvent imprécise du fait des erreurs de rappel ou d'un trouble du cycle menstruel. Cette étude transversale et descriptive avait pour objectif d’évaluer la fiabilité de la DDR dans la datation de la grossesse en pratique courante à Yaoundé.

**Méthodes:**

Etude transversale et descriptive réalisée dans deux hôpitaux universitaires de Yaoundé du 15 décembre 2012 au 15 avril 2013. La collecte des données était effectuée par un interrogatoire des femmes enceintes au cours de la consultation prénatale et l'examen de leur dossier médical. Les femmes enceintes présentant une complication du premier trimestre (menace d'avortement, grossesse arrêtée ou extra-utérine, grossesse molaire) et celles ne se rappelant pas de leur DDR ont été exclues de l’étude. Les données étaient saisies dans Epi-Data 3.1 et analysées dans le logiciel SPSS. 21.

**Résultats:**

Cinq cent huit femmes enceintes ont été enrôlées dans cette étude, 267(52,56%) d'entre elles avaient noté leur DDR sur un support tandis que 241(47,44%) utilisaient leur mémoire pour se rappeler leur DDR. Cent dix-sept (23,03%) femmes enceintes avait réalisé une échographie de datation du premier trimestre et parmi elles, 50 (42,70%) avaient une discordance des âges gestationnels théorique et échographique. Le rappel de la DDR par la mémoire (OR. 3,46; IC: 1,59-7,53), le cycle irrégulier (OR. 6,15; IC: 1,24-30,4) et le doute sur la DDR communiquée (OR. 31,06; IC: 3.95-244) étaient les facteurs significativement associés à la discordance des AG théorique et échographique.

**Conclusion:**

La DDR utilisée pour la datation de la grossesse en pratique courante à Yaoundé est fréquemment imprécise.

## Introduction

La détermination précise de l’âge gestationnel est d'une importance capitale dans la prise de nombreuses décisions obstétricales pendant la grossesse, l'accouchement et le post partum [1, 2].

Les cliniciens disposent de plusieurs moyens de datation de la grossesse parmi lesquels la date de dernières règles (DDR) communiquée par la femme enceinte, l’échographie de datation du premier trimestre et parfois une estimation clinique basée sur la grosseur de l'utérus, le moment de la perception des mouvements actifs f'taux et la hauteur utérine [3]. Cependant les méthodes d'estimation clinique de l’âge gestationnel sont reconnues comme étant suboptimales et peu fiables [3].

Selon L'Institut National pour la santé et l'Excellence clinique du Royaume Uni, une échographie de datation réalisée entre 10 et 13 semaines et 6jours de grossesse est considérée comme étant la méthode de choix pour la détermination de l’âge gestationnel, car pendant cette période le taux de variation de la croissance fœtale est faible [4]. Par contre, la datation de la grossesse basée sur la DDR est une méthode simple et pas couteuse. C'est une méthode universellement accessible et particulièrement dans les pays pauvres [4, 5]. Toutefois, une approche d'estimation de l’âge gestationnel basée uniquement sur la DDR peut être approximative dans certaines circonstances telles que, l'irrégularité ou les variations individuelles de la durée du cycle menstruel, l'aménorrhée pré-conceptionnelle au décours de l'utilisation de contraception hormonale, le saignement d'implantation en début de grossesse et les erreurs de mémorisation de la DDR par la femme enceinte [4, 6]. L'imprécision de la DDR pourrait alors conduire à une estimation erronée de l’âge de la grossesse et déboucher sur des diagnostics parfois inexacts de grossesse prolongée ou de travail prématuré entrainant la prise de décisions obstétricales inadaptées d'induction ou de tocolyse pouvant être à l'origine des complications materno-fœtales graves [3, 7].

La détermination précise de l’âge gestationnel est aussi essentielle dans l’évaluation de la croissance fœtale et le diagnostic de retard de croissance intra utérin [3].

Au Cameroun, la datation de la grossesse est généralement basée sur la DDR communiquée par la femme enceinte à la première visite prénatale et parfois, lorsque cela est possible, une échographie de datation peut être réalisée.

L'objectif de notre étude était d’évaluer la fiabilité de la DDR utilisée comme méthode de datation de la grossesse en pratique courante à Yaoundé, dans le but d'améliorer la prise en charge obstétricale dans notre milieu.

## Méthodes

Cette étude transversale et descriptive s'est déroulée dans les services de consultations prénatales de deux hôpitaux universitaires de Yaoundé: l'Hôpital Général de Yaoundé et le Centre Hospitalier et Universitaire de Yaoundé. Elle s’étalait sur une période de 4 mois du 15 Décembre 2012 au 15 Avril 2013. La population d’étude était constituée de toutes les femmes enceintes reçues en consultation prénatale dans ces hôpitaux pendant la période d’étude. Nous avions exclu les femmes enceintes non consentantes ou présentant une complication du premier trimestre de grossesse (menace d'avortement, grossesse arrêtée ou extra-utérine, grossesse molaire) et celles ne se rappelant pas de la date des dernières règles. La taille de l’échantillon de cette étude était estimée à 481 femmes. Elle était calculée à partir de la proportion (14%) de femmes enceintes qui doutaient de leur DDR estimée à partir d'un échantillon de 100 femmes enceintes enregistrées au début de cette étude.

La collecte des données était effectuée par interrogatoire des femmes enceintes au cours de la consultation prénatale et à partir dossiers médicaux. Pour chaque femme incluse dans notre étude, les variables étudiées étaient: âge de la femme; statut marital (célibataire ou mariée); niveau d'instruction; la date des dernières règles(en précisant le premier jour); l'irrégularité du cycle menstruel (considérée pour toute variation du cycle menstruel supérieure à 7 jours ou des cycles menstruels de plus de 35jours); la pratique d'une méthode de contraception hormonale avant la grossesse; le doute de la femme enceinte sur la DDR communiquée; la méthode utilisée pour le rappel de la DDR: notation sur un support (calendrier de poche, calendrier mural, agenda, ordinateur portable ou de bureau) ou la mémoire (personnelle de la femme, celle de son conjoint, ou par rapport à un événement); l’âge gestationnel théorique calculé à partir de la DDR; l’âge gestationnel échographique calculé à partir de la mesure de la longueur cranio-caudale de l'embryon sur une échographie de datation du premier trimestre réalisée entre 10 et 13 semaines 6 jours de grossesse par un personnel compétent (radiologue ou gynécologue/obstétricien).

Les données étaient saisies dans le logiciel Epi-Data 3.1. puis exportées vers le logiciel SPSS.21 pour l'analyse statistique.

Nous avons utilisé le test du Khi-deux pour la comparaison de deux variables. L'Odd ratio était calculé pour certaines variables pouvant être associées à la discordance des âges gestationnels théorique et échographique. Le seuil de significativité statistique était fixé à 5%. Nous avons distingué deux groupes de femmes en fonction de la concordance ou non entre les âges gestationnels théorique et échographique. Le groupe discordant était constitué de femmes enceintes dont la différence entre les âges gestationnels théorique et échographique du 1^er^ trimestre était de plus de 7 jours. Le groupe concordant comprenait les femmes enceintes dont la différence entre les âges gestationnels théorique et échographique était de 7jours ou moins. Par ailleurs, nous avions obtenu préalablement auprès du chef de la division médicale des hôpitaux concernés une autorisation administrative de réaliser cette étude.

## Résultats

Un total de 508 femmes enceintes a été enrôlé dans cette étude. L’âge médian des femmes était de 27,93 ans (16 ans - 44 ans) et 194 (38,18%) d'entre elles étaient nullipares tandis que 314 (61,81%) avaient déjà accouché au moins une fois. Les caractéristiques de notre population d’étude sont décrites dans le Tableau 1.

**Tableau 1:** Caractéristiques de la population d’étude

	Femmes enceintes (n = 508)	
Variables	Fréquence	%
**Age**		
<20ans	24	04,72
20-30ans	294	57,87
30-35ans	144	28,35
>35ans	46	09,06
**Statut matrimonial**		
Célibataire	277	54,52
Marié	231	45,47
**Niveau d'instruction**		
Universitaire	230	45,27
Secondaire	236	46,65
Primaire	40	07,69
Aucun	02	0,39
**Facteurs gynécologiques**		
Cycle menstruel irrégulier	41	08,07
Doute sur la DDR	82	16,14
DDR communiquée à partir du dernier jour	93	18,30
Contraception hormonale avant conception	18	03,54

### Caractéristiques de la population étudiée

Notre échantillon était constitué en majorité de femmes de la tranche d’âge de 20-30ans, concordant bien avec le profil de notre population composée en majorité de jeunes. Par ailleurs, 90% des femmes avaient un niveau d'instruction équivalent au moins au cycle secondaire, caractéristique d'une population urbaine des grandes métropoles africaines regorgeant des élèves, étudiants et travailleurs des secteurs publiques et privés.

### Moyens de rappel de la DDR utilisés par les femmes enceintes

Des 508 femmes enceintes incluses dans cette étude, 267 (52,56%) avaient noté leur DDR sur un support tandis que 241 (47.44%) d'entre elles utilisaient la mémoire pour se rappeler leur DDR. Le Tableau 2 montre la répartition des femmes enceintes selon le type de supports utilisé pour la notation de leur DDR. Les calendriers de poche et muraux étaient les supports de notation les plus utilisés par les femmes enceintes (86%).

**Tableau 2:** Répartition des femmes enceintes selon le type de supports utilisé pour la notation de leur DDR

Supports de notation de la DDR	Femmes enceintes (n= 267)	
	Fréquence	%
**Calendrier de poche**	188	70,41
**Calendrier mural**	41	15,36
**Agenda**	31	11,61
**Téléphone mobile**	6	2,25
**Ordinateur de bureau**	1	0,37

**Figure 1:**
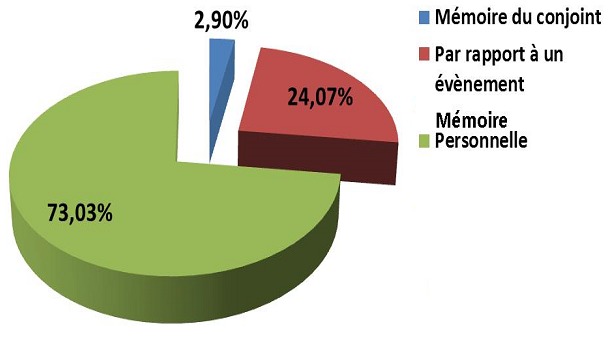
Répartition des femmes enceintes en fonction du type de mémoire utilisé pour se rappeler leur DDR

La Figure 1 représente la répartition des femmes enceintes selon le type de mémoire utilisé pour se rappeler la DDR. La majorité des femmes enceintes utilisant la mémoire pour se rappeler leur DDR faisait recours à leur capacité mnémonique personnelle (73,03%).

### Etude de la concordance entre les âges gestationnels théorique et échographique

**Figure 2:**
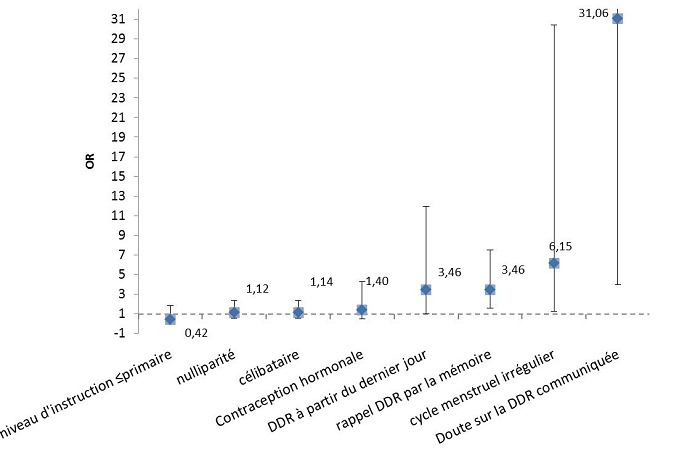
Graphique des Odds Ratios des variables associées à la discordance des âges gestationnels théorique et échographique

Un total de 117 (23,03%) femmes enceintes avait réalisé une échographie de datation du premier trimestre, 50 (42,70%) d'entre elles avaient une discordance (différence >7jours) des âges gestationnels théorique et échographique et représentaient le groupe discordant tandis que 67 (57,30%) femmes enceintes avaient plutôt une concordance (différence≥ 7jours) et représentaient le groupe concordant. Dans le groupe discordant, la moyenne de différence des âges gestationnels était de 9,5 jours, avec des extrêmes allant de 8 à 46 jours. Parmi les 117 femmes enceintes qui avaient réalisé une échographie du premier trimestre, 19 (16,24%) avaient une différence positive de la discordance des âges gestationnels théorique et échographique. Par contre 31 (26,50%) femmes avaient une différence négative de la discordance. L'utilisation de la mémoire pour le rappel de la DDR, OR. 3,46 (1,59-7,53); l'irrégularité du cycle menstruel, OR. 6,15 (1,24-30,4) et le doute sur la DDR communiquée OR.31,06(3,95-244) étaient significativement associés à la discordance des âges gestationnels théorique et échographique (Tableau 3, Figure 2).

**Tableau 3:** Etude de l'association entre certaines variables et la discordance entre les âges gestationnels théorique et échographique

Variables	Femmes enceintes				
	Groupe concordant	Groupe discordant			
	Fréquence (%)	Fréquence (%)	Total (%)	P	OR (95%CI)
Niveau d'instruction ≤ primaire	7(58,33)	5(41,66)	12(100)	0,520	0.42 (0,1-1,86)
Nulliparité	30(58,82)	21(41,17)	51(100)	0,071	1,12 (0,53-2,35)
Célibataire	29(52,72)	26(47,27)	55(100)	0,431	1,14 (0,55-2,37)
Contraception hormonale	7(46,66)	8(53,33)	15(100)	0,586	1,40 (0,46-4,27)
Rappel DDR par la mémoire	18 (39,10)	28 (60,90)	46 (100)	0,001	3,46 (1,59-7,53)
Doute sur la DDR communiquée	1 (5,90)	16 (94,10)	17 (100)	0,1	31,06 (3,95-244)
Cycle menstruel irrégulier	4 (30,80)	9 (62,20)	13 (100)	0,041	6,15 (1,24-30,4)
DDR à partir du dernier jour	4 (30,80)	9 (62,20)	13 (100)	0,041	3,46 (1-11,97)

## Discussion

Notre étude a retrouvé une fréquence élevée (42,07%) de discordance entre l’âge de la grossesse déterminé à partir de la DDR et celui estimé par l’échographie précoce du premier trimestre. Notre taux de discordance des âges gestationnels de 42,70% est proche du taux de 39,2% retrouvé dans l’étude ghanéenne [2] et plus élevé que le taux de 22% rapporté dans l’étude américaine [8]. Les différences socioculturelles entre les deux populations africaine et américaine pourraient expliquer cette différence de taux de discordance, sachant que les facteurs socioculturels pourraient influencer le choix des méthodes de rappel de la DDR et par conséquence avoir un impact sur la précision de celle-ci. D'autre part, le taux élevé de la discordance des âges gestationnels dans cette étude pourrait s'expliquer en partie par des erreurs de rappel de DDR au vu du taux élevé de la discordance des âges gestationnels théorique et échographique parmi les femmes qui utilisaient leur capacité mnémonique pour le rappel de leur DDR. Par ailleurs, cette étude a montré que le taux de discordance des âges gestationnels théorique et échographique était significativement plus élevé parmi les femmes enceintes qui utilisaient la mémoire pour se rappeler leur DDR (OR. 3,46 (1,59-7,53); celles qui avaient un cycle menstruel irrégulier OR. 6,15 (1,24-30,4) et celles qui avaient un doute sur leur DDR OR.31,06(3,95-244). Nos résultats corroborent les données de la littérature sur l'imprécision de la DDR dans la datation de la grossesse [2, 3, 6, 9–12].

D'autre part dans cette étude, 16,24% de femmes ayant réalisé une échographie de datation avaient une discordance positive des âges gestationnels (âge gestationnel théorique supérieur à l’âge gestationnel échographique) et 26,50% de femmes avaient une discordance négative (âge gestationnel théorique inférieur à l’âge gestationnel échographique). Lorsque l’âge gestationnel est surestimé, des décisions inappropriées peuvent être prises pour un dépassement de terme qui n'est en fait pas réel. Il en serait de même si l’âge gestationnel est sous-estimé, un accouchement à terme pourrait être considéré comme prématuré conduisant parfois à une tocolyse inappropriée. Le pourcentage de la discordance négative des âges gestationnels plus élevé dans cette étude contraste avec les résultats de certaines études antérieures [8, 11, 13] qui ont montré que l'utilisation de la DDR pour l'estimation de l’âge gestationnel avait plutôt tendance à surestimer les grossesses post termes. Cependant les résultats de notre étude concordent avec ceux des études ghanéenne [2] et de Yang et al. [14] qui ont trouvé comme nous un pourcentage plus élevé de la discordance négative.

Nous avons trouvé que 16,14% de femmes avaient un doute sur leur DDR. Ce résultat est proche de celui de Taipale et al. [15] qui ont trouvé que 21,2% de femmes étaient incertaines de leur DDR et en contraste avec celui de Campbell et al. [6] qui retrouvent dans leur étude 45% de femmes avec un doute sur leur DDR. Le pourcentage plus élevé de DDR douteuse dans l’étude de Campbell et al. peut s'expliquer par le fait que dans leur étude, le doute sur la DDR regroupait en plus de l'incertitude évoquée par la femme sur sa DDR considérée dans notre étude, les cycles irréguliers, la contraception hormonale avant la grossesse et le saignement en début de grossesse.

L’échographie est une méthode précise et utile pour l'estimation de l’âge gestationnel au premier trimestre de grossesse, lorsqu'elle fait partie du bilan anténatal de routine, elle peut énormément influencer la prise en charge obstétricale et améliorer les soins anténatals [3, 7, 12]. Seulement 17% des femmes enceintes avaient effectué une échographie de datation précoce dans cette étude réalisée en zone urbaine. On pourrait imaginer un taux encore plus faible en zone rurale où l'accessibilité à l’échographie est encore plus limitée. Ce faible taux de la pratique de l’échographie de datation dans notre milieu pourrait s'expliquer par plusieurs facteurs dont, la pauvreté (les femmes enceintes doivent pour la plupart payer de leur propre poche l’échographie en l'absence de la sécurité sociale); l'accessibilité géographique à l’échographie encore limitée (certains hôpitaux ne disposant pas toujours d'un échographe); le manque d'intérêt à l’échographie de datation manifesté par certains personnels de santé et la première visite prénatale généralement réalisée tardivement après la 16e semaine de grossesse. La principale limite de cette étude est que la population étudiée était principalement urbaine et non représentative de la population rurale. Par conséquence, il est difficile de généraliser ces résultats à toute la population.

## Conclusion

Cette étude a montré que l’âge de la grossesse estimé à partir de la DDR en pratique courante à Yaoundé était fréquemment (42,7%) discordant de celui estimé à partir de l’échographie de datation du premier trimestre, traduisant une imprécision de la DDR. Les principaux facteurs associés à cette imprécision de la DDR étaient l'utilisation de la mémoire pour le rappel de la DDR, l'irrégularité du cycle menstruel et le doute exprimé par la femme enceinte sur la DDR communiquée. Ainsi, les cliniciens dans notre milieu devraient être sensibilisés sur le risque d'erreur diagnostique de travail prématuré ou des grossesses post termes en l'absence d'une échographie précoce de datation de la grossesse. Aussi, nous recommandons au personnel de santé des pays pauvres d’éduquer et d'encourager les femmes en âge de procréer à noter leur DDR afin d'améliorer sa précision dans la datation de la grossesse.
